# Supplementary material for: Elimination of noise in optically rephased photon echoes
Source: Nat Commun. 2021 Jul 19;12:4378. doi: 10.1038/s41467-021-24679-4 (PMC8289862; doi:10.1038/s41467-021-24679-4)
Supplement: Supplementary file 1 — Supplementary Information [file 41467_2021_24679_MOESM1_ESM.pdf]

Supplementary Information for Elimination of Noise in Optically Rephased Photon Echoes

You-Zhi Ma,[1,2,*] Ming Jin,[1,2,*] Duo-Lun Chen,[1,2,*] Zong-Quan Zhou,[1,2,†] Chuan-Feng Li,[1,2,‡] and Guang-Can Guo[1,2]

[1] *CAS Key Laboratory of Quantum Information, University of Science and Technology of China, Hefei, 230026, China*

[2] *CAS Center For Excellence in Quantum Information and Quantum Physics, University of Science and Technology of China, Hefei, 230026, China*

(Dated: June 29, 2021)

Supplementary Note 1 - Experimental details

The laser (Toptica, TA-SHG) has a total power of 900 mW and is split into three parts: the input signal, the pump light for the memory crystal and the pump light for the filter crystal. The memory crystal and the filter crystal are placed in a cryostat (Montana Instruments) to achieve a sample temperature of approximately 3.5 K. The power of of the pump light is 320 mW and 50 μW for the memory crystal and the filter crystal, respectively. At the center of memory crystal, the signal beam and pump beam has a diameter of 60 μm and 180 μm, respectively. Two cycles of cold head vibrations provide a trigger signal for the experiment every 1.43 s which includes 400 ms for class cleaning, 200 ms for spin polarization, 200 ms for backpump, the storage process and some waiting times.

Absorption structures of the memory crystal and the filter crystal are shown in Supplementary Fig. 1a, where 0 MHz represents the center frequency of the signal pulse. We decrease the probe pulse to the photon-counting regime to determine the structure of the memory crystal and the filter crystal using a single-photon detector. The memory crystal has a 700-kHz absorption peak with an absorption depth $d = 0.6$ inside a empty pit with a 5-MHz bandwidth. The filter crystal has a transparency window of approximately 1 MHz with the background absorption depth approximately 6.6, when used in a double-pass configuration.

Given the low absorption of the memory crystal, which is a typical problem for Eu doped crystals, we can compare the efficiency performances of NLPE and atomic frequency comb (AFC) memories. Here we assume squarish comb peaks to optimize the AFC efficiency which can be calculated as [1, 2]

$$\eta = (d/F)^2 e^{-(d/F)} \sin^2(\pi/F)/(\pi/F)^2.$$

The maximal efficiency is obtained with a comb finesse $F = \pi/\arctan(2\pi/d)$. Given the experimental parameter $d = 0.6$, the comb finesse is set as the optimized value of 2.1 and the theoretically bounded efficiency of the two-level AFC is 2.7% for our experimental configurations. In practice, the efficiency of spin-wave AFC would be much lower than that of the optimal two-level AFC considered here, because of the imperfect spin-wave transfer, the spin dephasing and the imperfect comb structure. As a result, the storage efficiency of NLPE obtained in work is much larger than that can be obtained with AFC. This advantage is also reflected in the better performances in SNR and the spin-wave storage fidelity that we obtained here, as compared to that in previous demonstrations of AFC memories using Eu-based materials [3–5].

Photon counting histograms for storage of time-bin qubits are shown in Supplementary Fig. 2. For storage of states $|e\rangle$ and $|l\rangle$ (Supplementary Fig. 2a), the input signal is a truncated Gaussian pulse with a full width at half maximum of 2.6 μs, and the center of the pulse is $t_{0\mathrm{e}} = 4.7$ μs and $t_{0\mathrm{l}} = 8.7$ μs for $|e\rangle$ and $|l\rangle$, respectively. π_{35} with a pulse length of 3.75 μs and a frequency span of 3.4 MHz incidents at $t_1 = 12.6$ μs. π_{13} with a pulse length of 1.5 μs and a frequency span of 2 MHz incidents at $t_2 = 15.1$ μs. Then we wait for 13 μs to separate the fourth π pulse and the echo, the second π_{13} pulse and the second π_{35} pulse incident at $t_3 = 29.5$ μs and $t_4 = 32.0$ μs, respectively. The echo emits at $t_5 = t_4 + t_3$ - t_2 - t_1 + t_0, which is 38.5 μs and 42.5 μs for input of $|e\rangle$ and $|l\rangle$, respectively. Due to the extended storage time, the average storage efficiency for $|e\rangle$ and $|l\rangle$ drops to 3.4%. This efficiency is employed in calculation of the classical bound in Supplementary Fig. 2c. For storage of states $|e\rangle + |l\rangle$ (Supplementary Fig. 2b) and $|e\rangle + i\,|l\rangle$, a controlled relative phase is introduced between $|e\rangle$ and $|l\rangle$. π_{35} incident at $t_1 = 12.6$ μs. π_{13} incidents at $t_2 = 15.1$ μs. Then we wait for 13 μs to separate the fourth π pulse and the echo, the second π_{13} pulse and two $(\frac{\pi}{2})_{35}$ pulses incident at $t_3 = 29.5$ μs and $t_{4e} = 30.4$ μs, $t_{4l} = 34.4$ μs, respectively. $(\frac{\pi}{2})_{35}$ is square pulse with a pulse length of 0.8 μs and the center of the two $(\frac{\pi}{2})_{35}$ pulses separate 4 μs from each other, corresponding to the total storage time of 32.2 μs and 36.2 μs. The echo emits at $t_5 = t_4$ + t_3 - t_2 - t_1 + t_0, which is 36.9 μs, 40.9 μs and 44.9 μs for output bins $|ee\rangle$, $|el + le\rangle$ and $|ll\rangle$, respectively.

In Supplementary Fig. 2c, we present the theoretically expected fidelity for our NLPE memory. The calculations are based on current experimental parameters using a method following that presented in Ref. [6]. The interference measurements are realized by two $(\frac{\pi}{2})_{35}$ pulses rather than a single π pulse, therefore, the readout effi-

ciency for superposition states is slightly lower than that for eigenstates ($|e\rangle$ or $|l\rangle$) by a raio defined as α. We further define another parameter, μ_1, as the input photon number, which enables detecting an echo with a SNR of 1. In our experiment, the μ_1 parameter for measurements on the eigenstates is $\mu_{1\mathrm{p}} = 0.10 \pm 0.02$, and $\alpha = 1.2 \pm 0.2$. Then the theoretical prediction on the storage fidelity is given by [6]:

$$F_{\mathrm{T}} = \frac{1}{3}(\frac{\mu + \mu_{1\mathrm{p}}}{\mu + 2\mu_{1\mathrm{p}}}) + \frac{1}{3}(1 + \frac{\mu}{\mu + 2\alpha\mu_{1\mathrm{p}}}),$$

which surpasses the classical bound staring from an input μ of 0.25 photons per pulse, as shown in Supplementary Fig. 2c.

Supplementary Note 2 - Analytic treatment of NLPE

Definitions and Hamiltonian

Here we provide a theoretical model for descriptions of single-photon storage based on NLPE according to a complete quantum treatment for photon-atom interactions. We consider an ensemble of atoms with four energy levels $|\pm1/2\rangle_{\mathrm{g}}$, $|\pm3/2\rangle_{\mathrm{g}}$, $|\pm3/2\rangle_{\mathrm{e}}$ and $|\pm5/2\rangle_{\mathrm{e}}$. For convenience we denote the states as

$$|\pm1/2\rangle_{\mathrm{g}} = |1\rangle\,, \quad |\pm3/2\rangle_{\mathrm{g}} = |3\rangle\,,$$
$$|\pm3/2\rangle_{\mathrm{e}} = |\bar{3}\rangle\,, \quad |\pm5/2\rangle_{\mathrm{e}} = |\bar{5}\rangle\,.$$

Nearly all N atoms are set along the z direction with a length L. In the Heisenberg picture, the state of an atom j at the time t is described by a series of atomic field operators $\hat{\sigma}^{\mathrm{j}}_{\mu\nu}$, defined as [7]

$$\hat{\sigma}^{\mathrm{j}}_{\mu\nu} = |\mu\rangle_{\mathrm{jj}} \langle\nu|\, e^{i\omega_{\mu\nu}t}, \tag{1}$$

where μ, ν = 1, 3, $\bar{3}$, $\bar{5}$ corresponding to specific energy levels. $\omega_{\mu\nu}$ is the mean angular frequency gap between $|\mu\rangle$ and $|\nu\rangle$. That is, if E_μ and E_ν are the average energies of states $|\mu\rangle$ and $|\nu\rangle$ respectively, we have $\omega_{\mu\nu} = (E_\nu - E_\mu)/\hbar$.

The signal field resonant with the transition $|\pm1/2\rangle_{\mathrm{g}}$ $\leftrightarrow$ $|\pm5/2\rangle_{\mathrm{e}}$ propagates through the ensemble. At the position $\mathbf{r}$ it is described by [8]

$$\mathbf{\hat{E}}_{\mathrm{s}}(\mathbf{r}, t) = \frac{\hbar}{d_{1\bar{5}}} g\hat{\mathcal{E}}(z, t)\mathbf{e}_{\mathrm{s}} e^{i\mathbf{k}_{\mathrm{s}}\cdot\mathbf{r} - i\omega_{1\bar{5}}t} + H.c. \tag{2}$$

The control pulse resonant with the transition $|\mu\rangle - |\nu\rangle$ can be described by

$$\mathbf{E}^{\mu\nu}_{\mathrm{c}}(\mathbf{r}, t) = \frac{\hbar}{d_{\mu\nu}} \Omega_{\mu\nu}(z, t)\mathbf{e}_{\mathrm{c}} e^{i\mathbf{k}_{\mathrm{c}}\cdot\mathbf{r} - i\omega_{\mu\nu}t} + H.c. \tag{3}$$

Here $\hat{\mathcal{E}}$ is the dimensionless envelope of the signal and $\Omega_{\mu\nu}$ is the Rabi frequency of an optical pulse resonant with transition $|\mu\rangle \leftrightarrow |\nu\rangle$. $\mathbf{k}_{\mathrm{s}}$ and $\mathbf{k}_{\mathrm{c}}$ are the wave vectors of the signal and control fields, while $\mathbf{e}_{\mathrm{s}}$ and $\boldsymbol{e}_{\mathrm{c}}$ are their polarization vectors. $d_{\mu\nu}$ is the transition dipole moment of the transition $|\mu\rangle \leftrightarrow |\nu\rangle$ and g is the dipolar coupling strength.

In the rotating-wave approximation, the Hamiltonian of the system is $\hat{H} = \hat{H}_{\mathrm{l}} + \hat{H}_{\mathrm{a}} + \hat{V}_{\mathrm{s}} + \hat{V}_{\mathrm{c}}$ [7, 9]. Specifically, the Hamiltonian for the signal photons is given by [10]

$$\hat{H}_{\mathrm{l}} = \int_{\infty} d\mathbf{k}\hbar\omega \hat{a}^{\dagger}_{\mathbf{k}}\hat{a}_{\mathbf{k}}, \tag{4}$$

where $\hat{a}_{\mathbf{k}}$ is the annihilation operator of the wave vector $\mathbf{k}$. The Hamiltonian for the free atoms is given by

$$\hat{H}_{\mathrm{a}} = \sum_{\mathrm{j}} \sum_{\mu=3,\bar{3},\bar{5}} \hbar(\omega_{1\mu} + \delta^{\mathrm{j}}_{\mu})\hat{\sigma}^{\mathrm{j}}_{\mu\mu}, \tag{5}$$

where $\delta^{\mathrm{j}}_{\mu} = (E^{\mathrm{j}}_{\mu} - E_\mu)/\hbar$ is frequency detuning of the atom j to the average frequency for the state $|\mu\rangle$. The Hamiltonian for the electric-dipole interaction between atoms and the signal field is given by

$$\hat{V}_{\mathrm{s}} = -\hbar g \sum_{\mathrm{j}} \hat{\sigma}^{\mathrm{j}}_{\bar{5}1} \hat{\mathcal{E}}(z_{\mathrm{j}}, t) e^{i\mathbf{k}_{\mathrm{s}}\cdot\mathbf{r}_{\mathrm{j}}} + H.c, \tag{6}$$

while the Hamiltonian for the interaction between atoms and the control field is given by

$$\hat{V}_{\mathrm{c}} = -\hbar \sum_{\mathrm{j}} \sum_{\nu\neq\mu} \hat{\sigma}^{\mathrm{j}}_{\nu\mu} \Omega_{\mu\nu}(z_{\mathrm{j}}, t) e^{i\mathbf{k}_{\mathrm{c}}\cdot\mathbf{r}}. \tag{7}$$

In the mean field approximation, we introduce the average field operators [11]

$$\hat{\sigma}_{\mu\nu}(z, t; \delta) = \frac{1}{\mathcal{N}} \sum_{z_{\mathrm{j}}\in\mathcal{T}(z)} \sum_{\delta^{\mathrm{j}}\in\mathcal{Q}(\delta)} \hat{\sigma}^{\mathrm{j}}_{\mu\nu}(t). \tag{8}$$

Here $\mathcal{T}(z)$=$[z, z + dz]$ is a thin slice of the medium, large enough to contain a number of atoms but small enough compared to the wavelength of light. The quantity $\delta = \{\delta_1, \delta_3, \delta_{\bar{3}}, \delta_{\bar{5}}\}$ originates from the inhomogeneous broadening, c.f. Supplementary Fig. 1. $\delta^{\mathrm{j}} = \{\delta^{\mathrm{j}}_1, \delta^{\mathrm{j}}_3, \delta^{\mathrm{j}}_{\bar{3}}, \delta^{\mathrm{j}}_{\bar{5}}\}$ describes the unique energy structure of the atom j. $\mathcal{Q}(\delta)$ = $[\delta, \delta + d\delta]$ is a small frequency region compared with any inhomogeneous broadening but also contains a large number of atoms. $\mathcal{N}$ is the number of the atoms summed. By using average field operators, the summation over atoms in the Hamiltonian can be replaced by an integration as $\sum_{\mathrm{j}} \to \int n(z, \delta) dz d\delta$, where $n(z, \delta)$ is the spatial and frequency density of atoms and $d\delta$ is $d\delta_1 d\delta_3 d\delta_{\bar{3}} d\delta_{\bar{5}}$.

Equations of motion

We will use the Hamiltonian and several proper simplifications to obtain the equations of the evolution of the average values for operaters. To avoid clumsy notations,

we denote $\langle\hat{A}\rangle = A$ where $\hat{A}$ is an arbitrary operator we used.

The control pulses are short and strong, enabling us to divide the motions of the system according to whether it is under the optical control. When a control pulse is propagating through atoms, we neglect decay of the slow-varying atomic properties and the weak interaction between signal field and atoms, to simplify the Heisenberg equations for average values of operators as

$$\partial_t \sigma_{\mu\nu} = \frac{1}{i\hbar}\langle[\hat{\sigma}_{\mu\nu}, \hat{V}_{\rm c}]\rangle, \tag{9}$$

which is actually the form of the optical Bloch equations [7]. Here we also neglect the distortion or absorption of control pulses during their propagation through atoms.

In absence of control fields, atoms absorb or re-emit the signal field. Assume that the atoms are evenly distributed in space, the Heisenberg equations are reduced to

$$\begin{aligned}\partial_z \mathcal{E} + c^{-1}\partial_t \mathcal{E} &= igN\int_\infty d\delta\rho(\delta)\sigma_{1\bar{5}}e^{-i\mathbf{k}_{\rm s}\cdot\mathbf{z}},\\ \partial_t \sigma_{1\bar{5}} &= -(\gamma_{1\bar{5}} + i\delta_{1\bar{5}})\sigma_{1\bar{5}} + ig(\sigma_{11} - \sigma_{\bar{5}\bar{5}})\mathcal{E}e^{i\mathbf{k}_{\rm s}\cdot\mathbf{z}},\end{aligned} \tag{10}$$

$$\partial_t \sigma_{11} = -\partial_t \sigma_{\bar{5}\bar{5}} = -ig\sigma_{\bar{5}1}\mathcal{E}e^{i\mathbf{k}_{\rm s}\cdot\mathbf{z}} + ig\sigma_{1\bar{5}}\mathcal{E}^* e^{-i\mathbf{k}_{\rm s}\cdot\mathbf{z}}. \tag{11}$$

Here $\gamma_{1\bar{5}}$ is the decoherence rate of the optical coherence $\hat{\sigma}_{1\bar{5}}$. $\delta^{\rm j}_{\mu\nu} = \delta^{\rm j}_{\nu}$ - $\delta^{\rm j}_{\mu}$ is the atomic detuning for the transition $|\mu\rangle \leftrightarrow |\nu\rangle$. $\rho(\delta)$ characterizes the frequency density of the atomic system. Usually we neglect the term $c^{-1}\partial_t\mathcal{E}$ since the speed of light c is large enough when solving this equation.

For most of the storage time, atoms are allowed to undergo free evolution. The Heisenberg equations are reduced to

$$\partial_t \sigma_{\mu\nu} = \frac{1}{\hbar}\langle[\hat{\sigma}_{\mu\nu}, \hat{H}_{\rm a}]\rangle + \langle\mathcal{R}(\hat{\sigma}_{\mu\nu})\rangle. \tag{12}$$

$\mathcal{R}$ is the dissipator for the corresponding operator. These equations are in the similar form as the motion equations for damped oscillators.

Absorption

The complete state of the atomic system can be described by a matrix

$$(\sigma)(z,t;\delta) = \begin{pmatrix} \sigma_{11} & \sigma_{13} & \sigma_{1\bar{3}} & \sigma_{1\bar{5}} \\ \sigma_{31} & \sigma_{33} & \sigma_{3\bar{3}} & \sigma_{3\bar{5}} \\ \sigma_{\bar{3}1} & \sigma_{\bar{3}3} & \sigma_{\bar{3}\bar{3}} & \sigma_{\bar{3}\bar{5}} \\ \sigma_{\bar{5}1} & \sigma_{\bar{5}3} & \sigma_{\bar{5}\bar{3}} & \sigma_{\bar{5}\bar{5}} \end{pmatrix}. \tag{13}$$

After the preparation process at $t = t_0$, all atoms stay in the ground state $|\pm 1/2\rangle_{\rm g}$ as

$$(\sigma)(z,t_0;\delta) = \begin{pmatrix} 1 & 0 & 0 & 0 \\ 0 & 0 & 0 & 0 \\ 0 & 0 & 0 & 0 \\ 0 & 0 & 0 & 0 \end{pmatrix}. \tag{14}$$

Then a weak pulse resonant with $|\pm 1/2\rangle_{\rm g} \leftrightarrow |\pm 5/2\rangle_{\rm e}$ is sent to the medium as the signal. We denote $T_{\rm ij} = t_{\rm j} - t_{\rm i}$ (i, j = 1, 2,$\cdots$). The signal pulse is short enough with a duration $T_{\rm in}$ shorter than T_{01}. The absorption of the input signal can be solved by Supplementary Equation 10 as

$$\mathcal{E}(z,t) = \mathcal{E}_{\rm in}(z, t - t_0)e^{-\alpha z/2}, \tag{15}$$

where $\mathcal{E}_{\rm in}(t) = \mathcal{E}(0,\ t + t_0)$ represents the input signal field, nonzero only if $t \in [0,\ T_{\rm in}]$, and α is the absorption coefficient.

Right before we sent the first control pulse at t=t_1, atoms absorb the input signal adequately and the atomic state become

$$(\sigma)(z,t_1^-;\delta) = \begin{pmatrix} 1-e & 0 & 0 & \sigma_{\rm i}e^{\phi(t_1^-)} \\ 0 & 0 & 0 & 0 \\ 0 & 0 & 0 & 0 \\ \sigma_{\rm i}^* e^{\phi^*(t_1^-)} & 0 & 0 & e \end{pmatrix}. \tag{16}$$

Here e is a small population excitation of the state $|\bar{5}\rangle$ caused by the signal field, $e \ll 1$.

The initial coherence $\sigma_{1\bar{5}}(t_1^-) = \sigma_{\rm i}e^{\phi(t_1^-)}$ is excited by the signal field, where $\sigma_{\rm i}$ and $\phi(t_1^-)$ is solved by Supplementary Equation 10 as

$$\begin{aligned}\sigma_{\rm i}(z;\delta_{1\bar{5}}) &= ige^{-\alpha z/2}\int_\infty \mathcal{E}_{\rm in}(t')e^{(\gamma_{1\bar{5}}+i\delta_{1\bar{5}})t'}dt',\\ \phi(t_1^-) &= i\mathbf{k}_0\cdot\mathbf{z} - (\gamma_{1\bar{5}} + i\delta_{1\bar{5}})T_{01}.\end{aligned} \tag{17}$$

Here we have used $\sigma_{\bar{5}\bar{5}} \sim 0$ and $\sigma_{11} \sim 1$ for a weak input signal. We also assumed that the inhomogeneous broadening is large enough of transition $|\pm 1/2\rangle_{\rm g} \leftrightarrow |\pm 5/2\rangle_{\rm e}$ and atoms distribute evenly. The initial population excitation $e = \sigma_{\bar{5}\bar{5}}(t_1^-)$ can be solved by Supplementary Equation 11 as

$$e(z;\delta_{1\bar{5}}) = 2g^2 e^{-\alpha z}\left|\int_\infty \mathcal{E}_{\rm in}(t')e^{i\delta_{1\bar{5}}t'}dt'\right|^2, \tag{18}$$

where we have neglected the decoherence rate $\gamma_{1\bar{5}}$ since $1/\gamma_{1\bar{5}}$ is typically much longer than the considered time scale.

Optical control and free evolution

After the signal is absorbed, two pairs of π_{13} and π_{35} control pulses are applied to store and retrieve the signal. We will show how a general atomic system evolves with or without a control pulse. We assume that one sends a π_{35} pulse at time t to a general atomic system given as

$$(\sigma)(t^-) = \begin{pmatrix} s_{11} & s_{13} & s_{1\bar{3}} & s_{1\bar{5}} \\ s_{31} & s_{33} & s_{3\bar{3}} & s_{3\bar{5}} \\ s_{\bar{3}1} & s_{\bar{3}3} & s_{\bar{3}\bar{3}} & s_{\bar{3}\bar{5}} \\ s_{\bar{5}1} & s_{\bar{5}3} & s_{\bar{5}\bar{3}} & s_{\bar{5}\bar{5}} \end{pmatrix}, \tag{19}$$

where all $s_{\mu\nu}=\sigma_{\mu\nu}(t^-)$. The effect of π_{35} can be solved by Supplementary Equation 9 as

$$(\sigma)(t^+) = \pi_{35}^{\mathbf{k}}[(\sigma)(t^-)] = \begin{pmatrix} s_{11} & ie^{-i\mathbf{k}\cdot\mathbf{z}}s_{1\bar{5}} & s_{1\bar{3}} & ie^{i\mathbf{k}\cdot\mathbf{z}}s_{13} \\ -ie^{i\mathbf{k}\cdot\mathbf{z}}s_{\bar{5}1} & s_{\bar{5}\bar{5}} & -ie^{i\mathbf{k}\cdot\mathbf{z}}s_{\bar{5}\bar{3}} & e^{2i\mathbf{k}\cdot\mathbf{z}}s_{\bar{5}3} \\ s_{\bar{3}1} & ie^{-i\mathbf{k}\cdot\mathbf{z}}s_{\bar{3}\bar{5}} & s_{\bar{3}\bar{3}} & ie^{i\mathbf{k}\cdot\mathbf{z}}s_{\bar{3}3} \\ -ie^{-i\mathbf{k}\cdot\mathbf{z}}s_{31} & e^{-2i\mathbf{k}\cdot\mathbf{z}}s_{3\bar{5}} & -ie^{-i\mathbf{k}\cdot\mathbf{z}}s_{3\bar{3}} & s_{33} \end{pmatrix} \tag{20}$$

Similarly, the effect of the π_{13} on the same atomic system given in Supplementary Equation 19 is solved as

$$(\sigma)(t^+) = \pi_{13}^{\mathbf{k}}[(\sigma)(t^-)] = \begin{pmatrix} s_{\bar{3}\bar{3}} & -ie^{i\mathbf{k}\cdot\mathbf{z}}s_{\bar{3}3} & e^{2i\mathbf{k}\cdot\mathbf{z}}s_{\bar{3}1} & -ie^{i\mathbf{k}\cdot\mathbf{z}}s_{\bar{3}\bar{5}} \\ ie^{-i\mathbf{k}\cdot\mathbf{z}}s_{3\bar{3}} & s_{33} & ie^{i\mathbf{k}\cdot\mathbf{z}}s_{31} & s_{3\bar{5}} \\ e^{-2i\mathbf{k}\cdot\mathbf{z}}s_{1\bar{3}} & -ie^{-i\mathbf{k}\cdot\mathbf{z}}s_{13} & s_{11} & -ie^{-i\mathbf{k}\cdot\mathbf{z}}s_{1\bar{5}} \\ ie^{-i\mathbf{k}\cdot\mathbf{z}}s_{\bar{5}\bar{3}} & s_{\bar{5}3} & ie^{i\mathbf{k}\cdot\mathbf{z}}s_{\bar{5}1} & s_{\bar{5}\bar{5}} \end{pmatrix} \tag{21}$$

If there is no control pulse applied, the system in Supplementary Equation 19 will undergo free evolution $(\sigma)(t^- + T_{\text{ij}}) = E_{\text{ij}}[(\sigma)(t^-)]$, given specifically by Supplementary Equation 12 as

$$\begin{aligned} \sigma_{\mu\nu}(t^- + T_{\text{ij}}) &= s_{\nu\nu},\ (\mu = \nu) \\ \sigma_{\mu\nu}(t^- + T_{\text{ij}}) &= e^{-(\gamma_{\mu\nu}+i\omega_{\mu\nu})T_{\text{ij}}}s_{\mu\nu},\ (\mu \neq \nu) \end{aligned} \tag{22}$$

where $\gamma_{\mu\nu}=\gamma_{\nu\mu}$ is the decoherence rate of the transition $|\mu\rangle \leftrightarrow |\nu\rangle$. Here we only consider the decoherence term in the dissipator in Supplementary Equation 12 for a short storage time.

Silence and revival of the echoes

After the first pair of the control pulses are applied to the medium, the atomic state is given by

$$\begin{aligned} (\sigma)(t_2^+) &= \pi_{13}^{\mathbf{k}_2} \circ E_{12} \circ \pi_{35}^{\mathbf{k}_1}[(\sigma)(t_1^-)] \\ &= \begin{pmatrix} e & 0 & \sigma_{\text{i}}^* e^{\phi(t_2^+)} & 0 \\ 0 & 0 & 0 & 0 \\ \sigma_{\text{i}} e^{\phi^*(t_2^+)} & 0 & 1-e & 0 \\ 0 & 0 & 0 & 0 \end{pmatrix}, \end{aligned} \tag{23}$$

where function $\phi(t_2^+)$ is given by

$$\begin{aligned} \phi(t_2^+) =& i(-\mathbf{k}_0 + \mathbf{k}_1 + \mathbf{k}_2) \cdot \mathbf{z} \\ &- (\gamma_{1\bar{5}} - i\delta_{1\bar{5}})T_{01} - (\gamma_{13} + i\delta_{13})T_{12}. \end{aligned} \tag{24}$$

Atoms in such a state may emit an echo. According to Supplementary Equation 10 and Supplementary Equation 23, the generation of an echo can be described as

$$\begin{aligned} &\partial_z \mathcal{E} + \alpha/2\mathcal{E} = g^2 N e^{-\alpha z} e^{i(-\mathbf{k}_0+\mathbf{k}_1+\mathbf{k}_2-\mathbf{k}'_{\text{echo}})\cdot\mathbf{z}} \times \\ &\times \iint_\infty \rho(\delta)\{\mathcal{E}_{\text{in}}^*(t')e^{-\gamma_{1\bar{5}}(T_{01}+t-t_2-t')-\gamma_{13}T_{12}} \times \\ &\times e^{-i[\delta_{1\bar{5}}(t+t'-t_2-T_{01})+\delta_{13}T_{12}]}\}dt'd\delta. \end{aligned} \tag{25}$$

Since the $\mathcal{E}_{\text{in}}(t')$ is nonzero only if $t' \sim 0$, the atomic temporal phase is thus $\delta_{1\bar{5}}(t - t_2 - T_{01}) + \delta_{13}T_{12}$. The atomic detunning $\delta_{1\bar{5}}$ related to the inhomogeneous broadening of the optical transition which is the order of MHz. This is usually much larger than the detunning δ_{13} in the order of 10 kHz. Therefore, at $t = t_1 + t_2 - t_0$ the main phase $\delta_{1\bar{5}}(t-t_2-T_{01}) \sim 0$ and the temporal part of atoms are nearly rephased. A standard four-level echo [12] will emit if the spatial phase $(-\mathbf{k}_0 + \mathbf{k}_1 + \mathbf{k}_2 - \mathbf{k}'_{\text{echo}}) \cdot \mathbf{z} \sim 0$. Since the signal and the control pulse are nearly counter-propagating with each other, the phase matching condition

$$\mathbf{k}'_{\text{echo}} = -\mathbf{k}_0 + \mathbf{k}_1 + \mathbf{k}_2 \tag{26}$$

is not satisfied in the current experimental configuration. Therefore, the standard four-level echo which is emitted from population-inverted atoms, is silenced in NLPE.

Since the four-level echo is silenced, during $[t_2^+,\ t_3^-]$ atoms undergo free evolution. Then after the complete implementation of the NLPE protocol, the atomic state is given by

$$\begin{aligned} (\sigma)(t_4^+) =& \pi_{35}^{\mathbf{k}_4} \circ E_{34} \circ \pi_{13}^{\mathbf{k}_3} \circ E_{23}[(\sigma)(t_2^+)] \\ =& \begin{pmatrix} 1-e & 0 & 0 & \sigma_{\text{i}} e^{\phi(t_4^+)} \\ 0 & 0 & 0 & 0 \\ 0 & 0 & 0 & 0 \\ \sigma_{\text{i}}^* e^{\phi^*(t_4^+)} & 0 & 0 & e \end{pmatrix}, \end{aligned} \tag{27}$$

where $\phi(t_4^+)$ is given by

$$\begin{aligned} \phi(t_4^+) =& i(\mathbf{k}_0 - \mathbf{k}_1 - \mathbf{k}_2 + \mathbf{k}_3 + \mathbf{k}_4) \cdot \mathbf{z} \\ &- (\gamma_{13} + i\delta_{13})(T_{12} + T_{34}) - (\gamma_{3\bar{3}} - i\delta_{3\bar{3}})T_{23} \\ &- (\gamma_{1\bar{5}} + i\delta_{1\bar{5}})T_{01}. \end{aligned} \tag{28}$$

The atoms in this state may also emit an echo. Based on Supplementary Equation 27, we reduce Supplementary Equation 10 for echo generation to

$$\begin{aligned} &\partial_z \mathcal{E} + \alpha/2\mathcal{E} = -g^2 N e^{-\alpha z} e^{i(\mathbf{k}_0-\mathbf{k}_1-\mathbf{k}_2+\mathbf{k}_3+\mathbf{k}_4-\mathbf{k}_{\text{echo}})\cdot\mathbf{z}} \times \\ &\times \iint_\infty \rho(\delta)\{\mathcal{E}_{\text{in}}(t')e^{-\gamma_{13}(T_{12}+T_{34})-\gamma_{3\bar{3}}T_{23}-\gamma_{1\bar{5}}(T_{01}+t-t_4-t')} \times \\ &\times e^{-i[\delta_{13}T_{14}+\delta_{\bar{3}\bar{5}}T_{23}+\delta_{1\bar{5}}(t-t_4-t'+T_{01}-T_{23})]}\}dt'd\delta \end{aligned} \tag{29}$$

The phase matching condition for NLPE is thus

$$\mathbf{k}_{\text{echo}} = \mathbf{k}_0 - \mathbf{k}_1 - \mathbf{k}_2 + \mathbf{k}_3 + \mathbf{k}_4. \tag{30}$$

Therefore, $\mathbf{k}_{\text{echo}} = \mathbf{k}_0$ in our current experimental configuration. Similar as the analysis of four-level echo, since $\mathcal{E}_{\text{in}}(t')$ is nonzero only if $t' \sim 0$, the atomic temporal phase is thus $\delta_{1\bar{5}}(t - t_4 + T_{01} - T_{23}) + \delta_{13}T_{14} + \delta_{\bar{3}\bar{5}}T_{23}$ and $\delta_{1\bar{5}}$ is usually much larger than δ_{13} or $\delta_{\bar{3}\bar{5}}$. At $t = t_5 = t_0 - t_1 - t_2 + t_3 + t_4$ the main phase $\delta_{1\bar{5}}(t-t_4+T_{01}-T_{23}) \sim 0$. The atoms are nearly rephased in both time and space and the NEPE echo is emitted from a non-inverted atomic ensemble The small temporal phase $\delta_{13}T_{14}$ and $\delta_{\bar{3}\bar{5}}T_{23}$ can not be totally removed at $t = t_5$, leading to an incomplete rephasing at t_5. They are treated as the dephasing process in NLPE.

Output signal and efficiency

To calculate the output signal field, we need the specific form of frequency density $\rho(\delta)$. Here we assume that the inhomogeneous lineshapes of $|\pm1/2\rangle_{\mathrm{g}} \leftrightarrow |\pm3/2\rangle_{\mathrm{g}}$ and $|\pm3/2\rangle_{\mathrm{e}} \leftrightarrow |\pm5/2\rangle_{\mathrm{e}}$ are Gaussian functions and atoms evenly distribute on the broadening of $|\pm1/2\rangle_{\mathrm{g}} \leftrightarrow |\pm5/2\rangle_{\mathrm{e}}$. The frequency density of atoms then satisfies

$$\rho(\delta) = \frac{e^{-(\ln 2)\delta_{13}^2/\pi^2\Gamma_{13}^2}}{\Gamma_{13}\sqrt{\pi^3/\ln 2}} \frac{e^{-(\ln 2)\delta_{\bar{3}\bar{5}}^2/\pi^2\Gamma_{\bar{3}\bar{5}}^2}}{\Gamma_{\bar{3}\bar{5}}\sqrt{\pi^3/\ln 2}} \frac{\Theta(\Gamma_{1\bar{5}} - |\delta_{1\bar{5}}|/2\pi)}{\Gamma_{1\bar{5}}} \cdots \tag{31}$$

where $\Gamma_{\mu\nu}$ is the bandwidth of the inhomogeneous broadening of the transition $|\mu\rangle \leftrightarrow |\nu\rangle$ and Θ is the Heaviside-theta function. For a Gaussian lineshape we take the bandwidth as its full width at half maximum (FWHM).

For forward retrieval, the wave vector of the output echo $\mathbf{k}_{\mathrm{echo}}=\mathbf{k}_0$. The envelope of the output signal $\mathcal{E}_{\mathrm{out}}(t) = \mathcal{E}(L,t)$ can be solved by Supplementary Equation 29 as

$$\mathcal{E}_{\mathrm{out}}(t) = -de^{-d/2}e^{-\gamma_{13}(T_{12}+T_{34})-(\gamma_{3\bar{3}}+\gamma_{1\bar{5}})T_{23}} \times \\ \times e^{-\frac{\Gamma_{13}^2 T_{14}^2+\Gamma_{\bar{3}\bar{5}}^2 T_{23}^2}{4\ln 2/\pi^2}} \mathcal{E}_{\mathrm{in}}[t-(t_0-t_1-t_2+t_3+t_4)], \tag{32}$$

where $d=\alpha L$ is the optical depth. The maximal efficiency is d^2e^{-d} for forward retrieval. The imperfections of the rephasing π pulses can be included by multiplying a factor of $(\eta_{\mathrm{control}})^4$, assuming that these pulses have the same fidelity. The overall expression of the efficiency is given by

$$\eta_{\mathrm{NLPE}} = d^2e^{-d}(\eta_{\mathrm{control}})^4 e^{-\frac{\Gamma_{13}^2(t_4-t_1)^2+\Gamma_{\bar{3}\bar{5}}^2(t_3-t_2)^2}{2\ln 2/\pi^2}} \times \\ \times e^{-2\gamma_{13}(t_2+t_4-t_1-t_3)-2(\gamma_{3\bar{3}}+\gamma_{1\bar{5}})(t_3-t_2)}. \tag{33}$$

As we have mentioned before, the inhomogeneous broadening of the spin transitions $|\pm1/2\rangle_{\mathrm{g}} \leftrightarrow |\pm3/2\rangle_{\mathrm{g}}$ and $|\pm3/2\rangle_{\mathrm{e}} \leftrightarrow |\pm5/2\rangle_{\mathrm{e}}$ are treated as the dephasing in NEPE [13], characterized by Γ_{13} and $\Gamma_{\bar{3}\bar{5}}$. The spin decoherence rate γ_{13} is typically much smaller than other items and can be ignored. We further define an effective optical decoherence rate $\gamma = \gamma_{3\bar{3}} + \gamma_{1\bar{5}}$. Then the efficiency can be reduced to :

$$\eta_{\mathrm{NLPE}} = d^2e^{-d} \cdot (\eta_{\mathrm{control}})^4 \cdot \\ e^{-\frac{\Gamma_{13}^2(t_4-t_1)^2}{2\ln 2/\pi^2}} \cdot e^{-\frac{\Gamma_{\bar{3}\bar{5}}^2(t_3-t_2)^2}{2\ln 2/\pi^2}-2\gamma(t_3-t_2)}. \tag{34}$$

Supplementary Equation 34 provides a nice fit to our experimental data presented in Fig. 3 in the main text.

Phase compensation of CHS in NLPE

In our theoretical model, we only consider standard π pulses for simplicity, while CHS pulses are employed for robust control in the experiment. The CHS pulse is one kind of adiabatic rapid passage (ARP) pulse. It has been proved analytically and experimentally that a pair of identical ARPs are able to achieve perfect optical-to-spin coherence transformation, without introducing an extra phase which is different for atoms with different transition frequencies [14–16]. According to Ref. [15], the propagator of a pair of identical ARPs just adds a global phase compared with a pair of standard π pulse. Generally, for a three-level system including energy levels $|a\rangle$, $|b\rangle$ and $|c\rangle$, if there is coherence between transition $|a\rangle\leftrightarrow|b\rangle$, it can be completely rephased after one applies a pair of consecutive ARPs resonant with the transition $|a\rangle\leftrightarrow|c\rangle$ or the transition $|b\rangle\leftrightarrow|c\rangle$.

Based on on this conclusion, we can further prove that the ARPs used in NLPE memory will not introduce transition frequency dependent extra phase to the coherence. In NLPE, signal coherence is firstly generated between transition $|\pm1/2\rangle_{\mathrm{g}}\leftrightarrow|\pm5/2\rangle_{\mathrm{e}}$. Then the first single ARP resonant with transition $|\pm3/2\rangle_{\mathrm{g}}\leftrightarrow|\pm5/2\rangle_{\mathrm{e}}$ is applied, and the coherence is transferred to transition $|\pm3/2\rangle_{\mathrm{g}}\leftrightarrow|\pm1/2\rangle_{\mathrm{g}}$, attached with a transition frequency dependent extra phase generated by the first ARP. After that, a pair of identical ARPs resonant with transition $|\pm1/2\rangle_{\mathrm{g}}\leftrightarrow|\pm3/2\rangle_{\mathrm{e}}$ are applied. Applying the above-mentioned conclusion to the subsystem including energy levels $|\pm1/2\rangle_{\mathrm{g}}$, $|\pm3/2\rangle_{\mathrm{g}}$, $|\pm3/2\rangle_{\mathrm{e}}$, it is obvious that this pair of ARPs will not introduce any transition frequency dependent extra phase, so in the analysis of the extra phase we can neglect them. Since we have neglected the two ARPs resonant with transition $|\pm1/2\rangle_{\mathrm{g}}\leftrightarrow|\pm3/2\rangle_{\mathrm{e}}$, the first and last ARPs resonant with transition $|\pm3/2\rangle_{\mathrm{g}}\leftrightarrow|\pm5/2\rangle_{\mathrm{e}}$ then form one pair of identical ARPs. Applying the above-mentioned conclusion to the subsystem including energy levels $|\pm1/2\rangle_{\mathrm{g}}$, $|\pm3/2\rangle_{\mathrm{g}}$, $|\pm5/2\rangle_{\mathrm{e}}$, this pair of ARPs also generate no transition frequency dependent extra phase. As a result, the extra phase introduced by the CHS pulse is completely compensated in the NLPE protocol.

* These authors contributed equally
† zq_zhou@ustc.edu.cn
‡ cfli@ustc.edu.cn

[1] Bonarota, M., Ruggiero, J., Gouët, J. L. L. & Chanelière, T. Efficiency optimization for atomic frequency comb storage. *Phys. Rev. A* **81**, 033803 (2010).
[2] Jobez, P. *et al.* Towards highly multimode optical quantum memory for quantum repeaters. *Phys. Rev. A* **93**, 032327 (2016).
[3] Jobez, P. *et al.* Coherent spin control at the quantum level in an ensemble-based optical memory. *Phys. Rev. Lett.* **114**, 230502 (2015).
[4] Timoney, N., Usmani, I., Jobez, P., Afzelius, M. & Gisin, N. Single-photon-level optical storage in a solid-state spin-wave memory. *Phys. Rev. A* **88**, 022324 (2013).

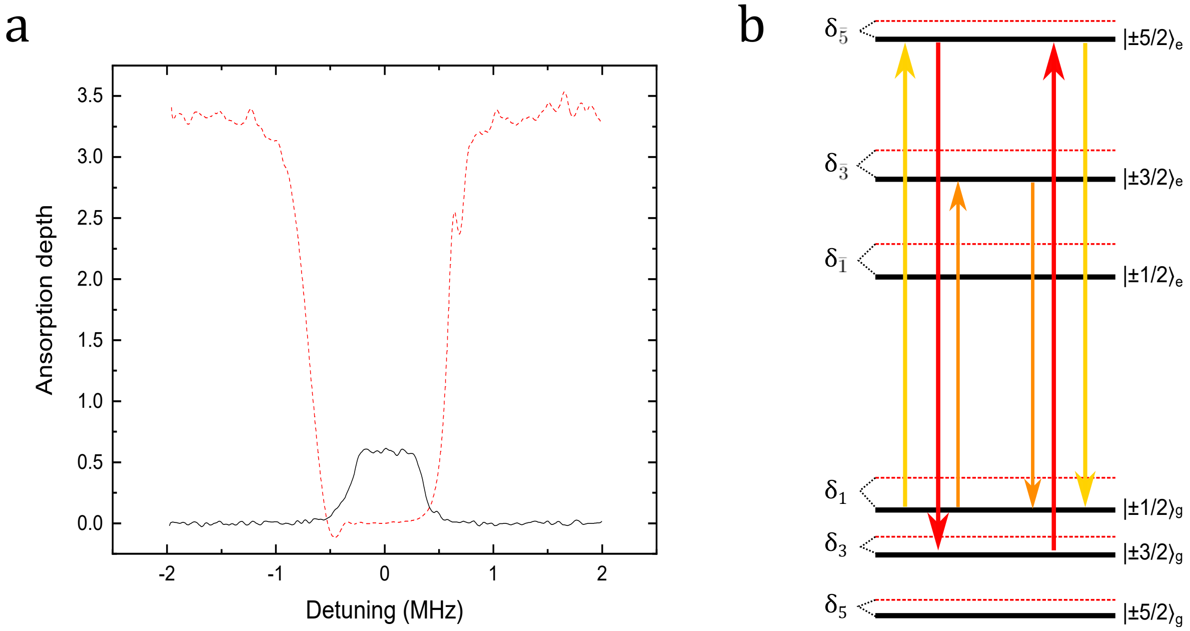

Supplementary Fig. 1. **Absorption structure and energy level diagram. a,** Absorption structure of the memory crystal (red dashed line) and the filter crystal (black solid line). Zero detuning corresponds to the frequency center of the signal pulse. **b,** The red dashed lines are energy levels of an arbitrary atom in the inhomogeneous broadening and the black solid lines are the energy levels in resonance with laser beams. δ represents the detuning of each transition of this atom.

[5] Laplane, C. *et al.* Multiplexed on-demand storage of polarization qubits in a crystal. *New Journal of Physics* **18**, 013006 (2015).

[6] Gündoğan, M., Ledingham, P. M., Kutluer, M., Kutlu. Mazzera & Riedmatten, H. d. Solid state spin-wave quantum memory for time-bin qubits. *Phys. Rev. Lett.* **114**, 230501 (2015).

[7] Cohen-Tannoudji, C., Dupont-Roc, J. & Grynberg, G. *Atom-Photon Interactions: Basic Processes and Applications* (1998).

[8] Loudon, R. *The Quantum Theory of Light* (2000).

[9] Scully, M. O. & Zubairy, M. S. *Quantum optics* (1999).

[10] Gorshkov, A. V., André, A., Lukin, M. D. & Sørensen, A. S. Photon storage in Λ-type optically dense atomic media. ii. free-space model. *Phys. Rev. A* **76**, 033805 (2007).

[11] Gorshkov, A. V., André, A., Lukin, M. D. & Sørensen, A. S. Photon storage in Λ-type optically dense atomic media. iii. effects of inhomogeneous broadening. *Phys. Rev. A* **76**, 033806 (2007).

[12] Beavan, S. E., Ledingham, P. M., Longdell, J. J. & Sellars, M. J. Photon echo without a free induction decay in a double-λ system. *Opt. Lett.* **36**, 1272–1274 (2011).

[13] Gündoğan, M., Mazzera, M., Ledingham, P. M., Cristiani, M. & de Riedmatten, H. Coherent storage of temporally multimode light using a spin-wave atomic frequency comb memory. *New Journal of Physics* **15**, 045012 (2013).

[14] Hwang, T. & Shaka, A. Water suppression that works. excitation sculpting using arbitrary wave-forms and pulsed-field gradients. *Journal of Magnetic Resonance, Series A* **112**, 275–279 (1995).

[15] Minář, J. c. v., Sangouard, N., Afzelius, M., de Riedmatten, H. & Gisin, N. Spin-wave storage using chirped control fields in atomic frequency comb-based quantum memory. *Phys. Rev. A* **82**, 042309 (2010).

[16] Pascual-Winter, M. F., Tongning, R.-C., Chanelière, T. & Gouët, J.-L. L. Securing coherence rephasing with a pair of adiabatic rapid passages. *New Journal of Physics* **15**, 055024 (2013).

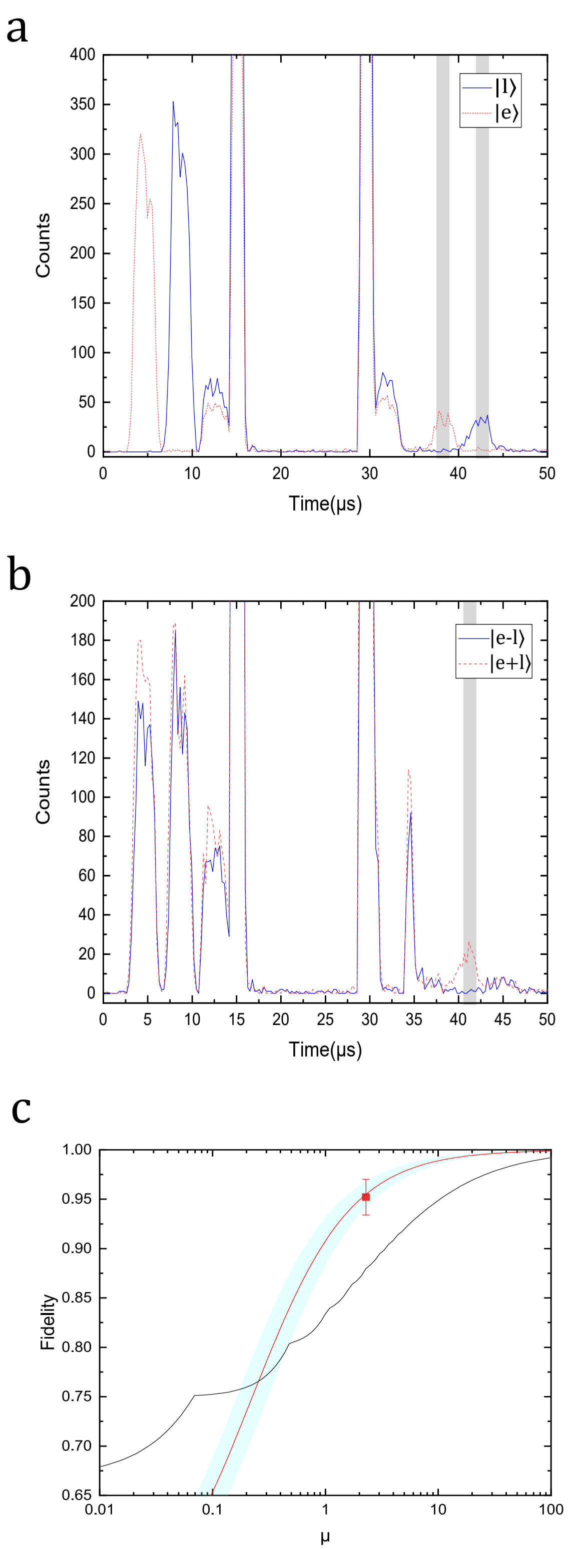

Supplementary Fig. 2. **Photon counting histogram for storage of time-bin qubits.** **a,** $|e\rangle$ and $|l\rangle$ with an average photon number of 2.29±0.06 photons per qubit. The red dashed and blue solid lines correspond to measurements with input $|e\rangle$ and $|l\rangle$, respectively. The data inside the shadowed area is employed for calculating fidelity. **b,** Photon counting histogram for storage of time-bin qubits $|e\rangle + |l\rangle$ with the same photon number per qubit. The red dashed line and the blue solid line correspond to the maximal interference and minimal interference obatined in the middle output bin, respectively. The bin size is 262 ns, and the experiments are repeated for 20000 trials. The peak at 34.4 μs is scattering noise from the second $(\frac{\pi}{2})_{35}$ pulse. **c,** The black line shows the classical bound which is the maximal fidelity that can be achieved using the classical measure-and-prepare strategy, taking into account the finite storage efficiency and the Poisson distribution of the photon source [6], with varying mean input photon number μ. The experimentally measured NLPE fidelity (95.2% $\pm$ 1.8%) for $\mu = 2.29$ is shown with the red square and the red error bar is 1 standard deviation of the fidelity. The red dashed line is the theoretical prediction on the storage fidelity based on the current experimental configurations and blue shaded area being the 1 standard deviation of the error in this prediction. The memory process remains in the quantum regime for $\mu > 0.25$.
